# High Cellular Monocyte Activation in People Living With Human Immunodeficiency Virus on Combination Antiretroviral Therapy and Lifestyle-Matched Controls Is Associated With Greater Inflammation in Cerebrospinal Fluid

**DOI:** 10.1093/ofid/ofx108

**Published:** 2017-05-25

**Authors:** Thijs Booiman, Ferdinand W. Wit, Irma Maurer, Davide De Francesco, Caroline A. Sabin, Agnes M. Harskamp, Maria Prins, Paolo Garagnani, Chiara Pirazzini, Claudio Franceschi, Dietmar Fuchs, Magnus Gisslén, Alan Winston, Peter Reiss, Neeltje A. Kootstra, On Behalf of the Comorbidity in Relation to AIDS (COBRA) Collaboration, P. Reiss, F. W. N. M. Wit, J. Schouten, K. W. Kooij, R. A. van Zoest, B. C. Elsenga, F. R. Janssen, M. Heidenrijk, W. Zikkenheiner, M. van der Valk, N. A. Kootstra, T. Booiman, A. M. Harskamp-Holwerda, B. Boeser-Nunnink, I. Maurer, M. M. Mangas Ruiz, A. F. Girigorie, J. Villaudy, E. Frankin, A. Pasternak, B. Berkhout, T. van der Kuyl, P. Portegies, B. A. Schmand, G. J. Geurtsen, J. A. ter Stege, M. Klein Twennaar, C. B. L. M. Majoie, M. W. A. Caan, T. Su, K. Weijer, P. H. L. T. Bisschop, A. Kalsbeek, M. Wezel, I. Visser, H. G. Ruhé, C. Franceschi, P. Garagnani, C. Pirazzini, M. Capri, F. Dall’Olio, M. Chiricolo, S. Salvioli, J. Hoeijmakers, J. Pothof, M. Prins, M. Martens, S. Moll, J. Berkel, M. Totté, S. Kovalev, M. Gisslén, D. Fuchs, H. Zetterberg, A. Winston, J. Underwood, L. McDonald, M. Stott, K. Legg, A. Lovell, O. Erlwein, N. Doyle, C. Kingsley, D. J. Sharp, R. Leech, J. H. Cole, S. Zaheri, M. M. J. Hillebregt, Y. M. C. Ruijs, D. P. Benschop, D. Burger, M. de Graaff-Teulen, G. Guaraldi, A. Bürkle, T. Sindlinger, M. Moreno-Villanueva, A. Keller, C. Sabin, D. de Francesco, C. Libert, S. Dewaele

**Affiliations:** 1 Department of Experimental Immunology and; 2 Department of Global Health and Division of Infectious Disease, Academic Medical Center, University of Amsterdam, Netherlands; 3 Amsterdam Institute for Global Health and Development, Netherlands; 4 Department of Infection and Population Health, University College London, United Kingdom; 5 Public Health Service, Amsterdam, Netherlands; 6 Department of Experimental, Diagnostic and Specialty Medicine, Alma Mater Studiorum Universita di Bologna, Italy; 7 Division of Biological Chemistry, Biocenter Innsbruck Medical University Center for Chemistry and Biomedicine, Austria; 8 Institute of Biomedicine, Department of Infectious Diseases, the Sahlgrenska Academy at the University of Gothenburg, Sweden; 9 Imperial College of Science, Technology and Medicine, London, United Kingdom; 10 HIV Monitoring Foundation, Amsterdam, Netherlands; and; 11 Istituto di Ricovero e Cura a Carattere Scientifico, Institute of Neurological Sciences of Bologna, Bellaria Hospital, Italy; 12 Academisch Medisch Centrum, Universiteit van Amsterdam - Department of Global Health and Amsterdam Institute for Global Health and Development; 13 Division of Infectious Diseases; 14 Department of Experimental Immunology; 15 Department of Medical Microbiology; 16 Department of Neurology; 17 Department of Radiology; 18 Department of Cell Biology; 19 Division of Endocrinology and Metabolism; 20 Department of Experimental Neuroendocrinology; 21 Department of Ophthalmology; 22 Department of Psychiatry; 23 Alma Mater Studiorum Universita di Bologna - Department of Experimental, Diagnostic and Specialty Medicine; 24 Erasmus Universitair Medisch Centrum Rotterdam - Department of Genetics; 25 GGD Amsterdam/Public Health Service Amsterdam - Cluster of Infectious Diseases, Research Department; 26 University of Gothenburg; 27 Imperial College of Science, Technology and Medicine - Department of Medicine, Division of Infectious Diseases; 28 Department of Medicine, Division of Brain Sciences, The Computational, Cognitive and Clinical Neuroimaging Laboratory; 29 Stichting HIV Monitoring; 30 Stichting Katholieke Universiteit Nijmegen; 31 Università Degli Studi di Modena e Reggio Emilia - Department of Medical and Surgical Sciences for Children and Adults; 32 Universität Konstanz - Department of Biology; 33 University College London - Research Department of Infection and Population Health; 34 Vlaams Instituut voor Biotechnologie - Inflammation Research Center

**Keywords:** CSF, HIV, immune activation, inflammation, monocyte

## Abstract

**Background:**

Increased monocyte activation and intestinal damage have been shown to be predictive for the increased morbidity and mortality observed in treated people living with human immunodeficiency virus (PLHIV).

**Methods:**

A cross-sectional analysis of cellular and soluble markers of monocyte activation, coagulation, intestinal damage, and inflammation in plasma and cerebrospinal fluid (CSF) of PLHIV with suppressed plasma viremia on combination antiretroviral therapy and age and demographically comparable HIV-negative individuals participating in the Comorbidity in Relation to AIDS (COBRA) cohort and, where appropriate, age-matched blood bank donors (BBD).

**Results:**

People living with HIV, HIV-negative individuals, and BBD had comparable percentages of classical, intermediate, and nonclassical monocytes. Expression of CD163, CD32, CD64, HLA-DR, CD38, CD40, CD86, CD91, CD11c, and CX3CR1 on monocytes did not differ between PLHIV and HIV-negative individuals, but it differed significantly from BBD. Principal component analysis revealed that 57.5% of PLHIV and 62.5% of HIV-negative individuals had a high monocyte activation profile compared with 2.9% of BBD. Cellular monocyte activation in the COBRA cohort was strongly associated with soluble markers of monocyte activation and inflammation in the CSF.

**Conclusions:**

People living with HIV and HIV-negative COBRA participants had high levels of cellular monocyte activation compared with age-matched BBD. High monocyte activation was predictive for inflammation in the CSF.

Successfully treated people living with human immunodeficiency virus (PLHIV) are reported to have higher rates of age- associated noncommunicable diseases (AANCCs) and a shorter average life expectancy compared with uninfected persons of the same age [1–3]. In addition to a higher prevalence of traditional risk factors for these AANCCs in PLHIV, combination antiretroviral therapy (cART) toxicity, chronic immune activation, and immune dysfunction have all been suggested to contribute to their development [4]. Several mechanisms have been proposed to explain the reported heightened inflammatory state observed in PLHIV, including (residual) HIV replication, HIV-mediated increased gut permeability and gut microbial translocation, coinfections with other viruses (ie, cytomegalovirus [CMV], hepatitis B virus [HBV], and hepatitis C virus [HCV]), and loss of immune regulatory responses [4–6]. Although cART reduces chronic immune activation, there remains evidence of continued high levels of T-cell activation [7, 8], cellular monocyte activation [9, 10], soluble monocyte activation markers [9, 11, 12], and inflammation [12–14] in PLHIV with suppressed plasma viremia on treatment. Furthermore, PLHIV were also shown to have persistently elevated concentrations of monocyte activation and inflammation in the cerebrospinal fluid (CSF) despite cART [15–17]. It is interesting to note that plasma markers of monocyte activation, inflammation, and intestinal damage (I-FABP), but not T-cell activation, have been found to be strong predictors of morbidity and mortality in treated PLHIV [18–20], suggesting that monocyte activation may be relevant to the pathogenesis underlying the increased morbidity and mortality that is seen in treated PLHIV. In this study, we analyzed cellular and soluble markers of monocyte activation, coagulation, intestinal damage, and inflammation in plasma and CSF of PLHIV on suppressive cART participating in the European Commission-funded Comorbidity in Relation to AIDS (COBRA) cohort study. These results were compared with those seen in a group of demographically comparable HIV-negative COBRA participants and, where appropriate, with age-matched blood bank donors (BBD). Furthermore, we analyzed the relation between markers of cellular monocyte activation and soluble markers of monocyte activation, intestinal damage, and inflammation in plasma and CSF of the PLHIV and HIV-negative COBRA participants.

## MATERIALS AND METHODS

### Subjects

The COBRA study included 134 PLHIV on cART and 79 demographically and lifestyle comparable HIV-negative control subjects from sites in London and Amsterdam in a detailed, prospective evaluation of the impact of HIV infection on the prevalence, incidence, and age at onset of AANCC. Exclusion criteria were as follows: age under 45 years (50 years for London participants); self-reported current intravenous drug use (in the past 6 months); daily use of recreational drugs (with the exception of cannabis); excess alcohol intake (>48 units per week); (history of) confounding neurological diseases; severe head injury (loss of consciousness for >30 minutes); infections or tumors involving the central nervous system ([CNS] including acquired immune deficiency syndrome-defining illnesses); current major depression (Patient Health Questionnaire-9 questionnaire score ≥15); self-reported current intravenous drug use in the past 6 months; daily use of recreational drugs (with the exception of cannabis); excess alcohol consumption (>48 units per week); severe psychiatric disorders; insufficient command of the Dutch/English language; and a contraindication to magnetic resonance imaging or lumbar puncture [50, 51]. All PLHIV were required to be on cART and to have had undetectable plasma HIV ribonucleic acid ([RNA] <50 copies/mL) for ≥12 months before enrollment. For the present immunological substudy, 40 PLHIV and 40 HIV-negative control participants were randomly selected with equal numbers in each of the following age groups: 45–50, 51–55, 56–60, 61–65, 66–70, and 70+, except for the oldest age category in which only a few individuals were available. Materials from 35 BBD were obtained from the Dutch national blood bank in Amsterdam, the Netherlands (www.sanquin.nl). Blood bank donors (median age, 58 years; interquartile range [IQR], 52–65) were matched for age with the PLHIV (median age, 58.5 years; IQR, 53–63) and HIV-negative COBRA participants (median age, 59 years; IQR, 53–64) and were selected in such a way that the different COBRA age categories (other than the 70+ category) were equally represented. Blood bank donors from the Netherlands were actively screened for HIV, HBV, HCV, syphilis, and human T-lymphotropic virus (HTLV) infection. Potential BBD were excluded from blood donation if they were determined to be at high risk of bloodborne infections (based on a questionnaire regarding general and sexual health, medication use, sexual risk behavior, and travel (https://www.sanquin.nl/en/give-blood/).

### Ethics Statement

This study was conducted in accordance with the ethical principles set out in the declaration of Helsinki and was approved by the institutional review board of the Academic Medical Center, the UK Research Ethics Committee (Stanmore, England, reference number 13/LO/0584), and the Ethics Advisory Body of the Sanquin Blood Supply Foundation in Amsterdam. Written informed consent was obtained from all participants.

### Monocyte and T-Cell Phenotyping and Flow Cytometry

Cryopreserved peripheral blood mononuclear cells (PBMCs) from COBRA participants and BBD were used for immune phenotyping. The PBMCs were thawed and subsequently stained with monoclonal antibodies (mAbs) for 30 minutes at 4°C in the dark, to determine expression of different surface molecules. The following directly conjugated mAbs were used for cell surface marker staining: CD14 PE-Cy7, CD16 eFluor 450, CD32 PerCP-eFluor 710, and CD11c APC (eBioscience, San Diego, CA); CD163 AlexaFluor 488 and CD86 PerCP (R&D Systems, Minneapolis, MN); CX3CR1 PerCP-Cy5.5 (BioLegend, San Diego, CA); HLA-DR V500, CD3 V500, CD4 PE-Cy7, HLA-DR fluorescein isothiocyanate (FITC), and CD38 PE (BD Biosiences, San Jose, CA); CD38 PE and CD91 PE (BD); and CD40 APC-H7, CD64 APC-H7, and CD8 Pacific Blue (BD Pharmingen, San Diego, CA). Fluorescence was measured with the FACS Canto II (BD Biosciences). The proportion of cells and the mean fluorescence intensity (MFI) of the markers was determined using FlowJo 7.6 (TreeStar, Ashland, OR).

### Soluble Markers in Plasma and Cerebrospinal Fluid

D-dimer and high-sensitivity C-reactive protein (CRP) concentrations were determined in fresh plasma samples using immunoturbidimetry (Sysmex CA-7000 [Siemens, Munich, Germany] and Cobas c702 [Roche Diagnostics, Risch-Rotkreuz, Switzerland]). I-FABP, sCD14, and sCD163 concentrations were determined in plasma and/or CSF samples stored at −80°C using enzyme-linked immunosorbent assay (ELISA) (I-FABP, CD14, and CD163 DuoSet ELISAs; R&D Systems). Soluble (s)CD16 concentrations were determined in plasma samples stored at −80°C by sandwich ELISA using mouse immunoglobulin (Ig)G1 antihuman CD16 3G8 mAb (BD Pharmingen) as capture antibody, FITC-conjugated mouse IgG antihuman CD16 DJ130c mAb (Dako) as detection antibody, and sheep IgG (Fab fragment) anti-FITC conjugated with horseradish peroxidase (Roche) as enzyme-linked secondary antibody, as described elsewhere [52]. Neopterin concentrations were measured in plasma and CSF stored at −80°C by ELISA (BRAHMS Diagnostics/Thermo Fisher, Henningsdorf, Berlin, Germany) [53]. Tryptophan and kynurenine concentrations were determined in plasma and CSF stored at −80°C by high-performance liquid chromatography [54]. The ratios of tryptophan/kynurenine were calculated as indexes of tryptophan breakdown, respectively. Tumor necrosis factor (TNF)α, interferon-γ-inducible protein 10 (IP-10)/CXCL10, macrophage inflammatory protein (MIP)1α/CCL3, interleukin (IL)-6, monocyte chemoattractant protein (MCP)1/CCL2, MIG/CXCL9, and RANTES/CCL5 concentrations were analyzed in plasma and CSF stored at −80°C by human magnetic luminex screening assay (LXSAHM-1 and LXSAHM-6; R&D Systems).

Cytomegalovirus total antibody titers were measured by ELISA-VIDITEST anti-CMV-IgG and IgG avidity (VIDIA, Praha, Czech Republic) according to the manufacturer’s instruction. For quantification, a standard curve was prepared by serial dilution of plasma from a known CMV-seropositive individual.

### Viral Load in Plasma and Cerebrospinal Fluid

Cerebrospinal fluid and plasma HIV RNA copy number was measured in stored CSF (−80°C) and fresh plasma samples using the Abbott RealTime M2000 assay (Abbot, Chicago, IL) with a lower limit of detection of 40 copies/mL.

### Statistical Analysis

Differences in subject characteristics between groups were assessed using Student’s *t* test, Mann-Whitney *U* test, or Pearson χ^2^ test, as appropriate. Significant differences of immunological markers between PLHIV, HIV-negative COBRA participants, and BBD were evaluated using multivariable linear regression adjusting for age and gender because some of the cellular markers are influenced by age and gender [55, 56]. Some outcomes were log_10_ transformed to attain normality as indicated. No formal Bonferroni correction was applied to *P* values due to its conservative nature when many tests are performed. Instead, principal components analysis (PCA) was performed to reduce the dimension of the dataset by allowing us to identify 1 or more linear combinations of expression markers, which together explain a reasonable proportion of the variation observed in the PLHIV, HIV-negative COBRA participants, and BBD. Therefore, our primary analysis focuses on the comparison of these linear combinations between groups. Principal components analysis was performed on the normalized MFIs of CD163, CD32, CD64, CD38, HLA-DR, CD40, CD86, CD91, CD11c, and CX3CR1 on the classical, intermediate, and nonclassical monocyte subsets. Analyses were performed in IBM SPSS Statistics for Windows version 23 (IBM, Armonk, NY), RStudio version 0.99.903 (RStudio, Inc., Boston, MA), and GraphPad Prism 6 (GraphPad, La Jolla, CA).

## RESULTS

### Baseline Characteristics of Comorbidity in Relation to AIDS Participants and Blood Bank Donors

People living with HIV and HIV-negative controls had a median age of 58.5 (IQR, 53–63) and 59 (IQR, 53–63) years, respectively. People living with HIV had been diagnosed with HIV for a median of 13.9 (IQR, 9.1–18.7) years, had been on cART for a median of 12.2 (IQR, 7.9–16.9) years, and had spent a median of 8.0 (IQR, 5.3–10.9) years with undetectable plasma HIV RNA (Table 1). All PLHIV had undetectable HIV RNA in CSF. Although the percentage of males did not differ significantly between the PLHIV and HIV-negative COBRA participants, it was significantly lower in the group of BBD. Despite long-term suppression of HIV-replication by cART, PLHIV in COBRA exhibited incomplete CD4^+^ T-cell restoration and greater CD8^+^ T-cell counts compared with HIV-negative participants, as reflected by lower CD4 counts, higher CD8 counts, and an inverted CD4/CD8 T-cell ratio (Table 1).

**Table 1.** Baseline Characteristics PLHIV, HIV-Negative COBRA Participants, and Blood Bank Donors

Baseline Characteristics	PLHIV *n* = 40	HIV Negative *n* = 40	PLHIV vs HIV Negative	BBD *n* = 35	PLHIV vs BBD	HIV Negative vs BBD
	n (%) or Median (IQR)	n (%) or Median (IQR)	*P* Value^a^	n (%) or Median (IQR)	*P* Value^a^	*P* Value^a^
Age (years)	58.5 (53.5–63.5)	59.0 (53.0–64.5)	.9	58 (52.0–65.0)	.5	.5
Male sex	36 (90.0%)	37 (92.5%)	.7	18 (51.4%)	**<.001**	**<.001**
African descent	5 (12.5%)	1 (2.5%)	.09	n.a.		
MSM	32 (80.0%)	30 (75.0%)	.8	n.a.		
CMV	38 (95.0%)	31 (77.5%)	**.02**	8 (22.9%)	**<.001**	**<.001**
Alcohol consumption (units/week)	7.7 (1–9)	5.6 (1–8)	.3	n.a.		
Recreational drug-use in past 6 months	6 (15%)	13 (33%)	.1	n.a.		
Smoking status			.3			
Never smoked	12 (30%)	10 (25%)		n.a.		
Ex-smoker	15 (37.5%)	22 (55%)		n.a.		
Current smoker	13 (32.5%)	8 (20%)		n.a.		
HBV	21 (52.5%)	7 (17.5%)	**.001**	n.a.		
Chronic HBV	3 (7.5%)	0 (0%)	.07	n.a.		
HCV	6 (15%)	0 (0%)	**.01**	0 (0%)	**.02**	n.d.
Chronic HCV	1 (2.5%)	0 (0%)	.3	n.a		
CD4 counts, cells/µL	589 (470–800)	961 (759–1233)	**<.001**	n.a.		
CD8 counts, cells/µL	762 (636–1029)	488 (364–621)	**<.001**	n.a.		
CD4/CD8 ratio	0.80 (0.61–1.13)	1.95 (1.33–2.83)	**<.001**	n.a.		
CD4 nadir, cells/µL	180 (60–180)					
Years since HIV diagnosis	13.9 (9.1–18.7)					
Years since start cART	12.2 (7.9–16.9)					
Years undetectable plasma HIV RNA (<200 c/mL)^b^	8.0 (5.3–10.9)					
Undetectable CSF HIV RNA (<40 c/mL)	40 (100%)					

Abbreviations: BBD, blood bank donors; cART, combination antiretroviral therapy; CMV, cytomegalovirus; CSF, cerebrospinal fluid; HBV, hepatitis B virus; HCV, hepatitis C virus; HIV, human immunodeficiency virus; IQR, interquartile range; MSM, men who have sex with men; n.a., not available; n.d., not determined; PLHIV, persons living with HIV; RNA, ribonucleic acid. Significant P values are depicted in bold.

^a^ *P* values calculated using Student’s *t* test, Mann-Whitney *U* test, or χ^2^ test or Wilcoxon’s rank-sum test where applicable.

^b^The threshold was set at 200 c/mL to exclude incidental viral blips from the period in which plasma viral load was detectable.

### Monocyte Activation in People Living With Human Immunodeficiency Virus (HIV) and HIV-Negative Comorbidity in Relation to AIDS Participants and Blood Bank Donors

**Figure 1.**
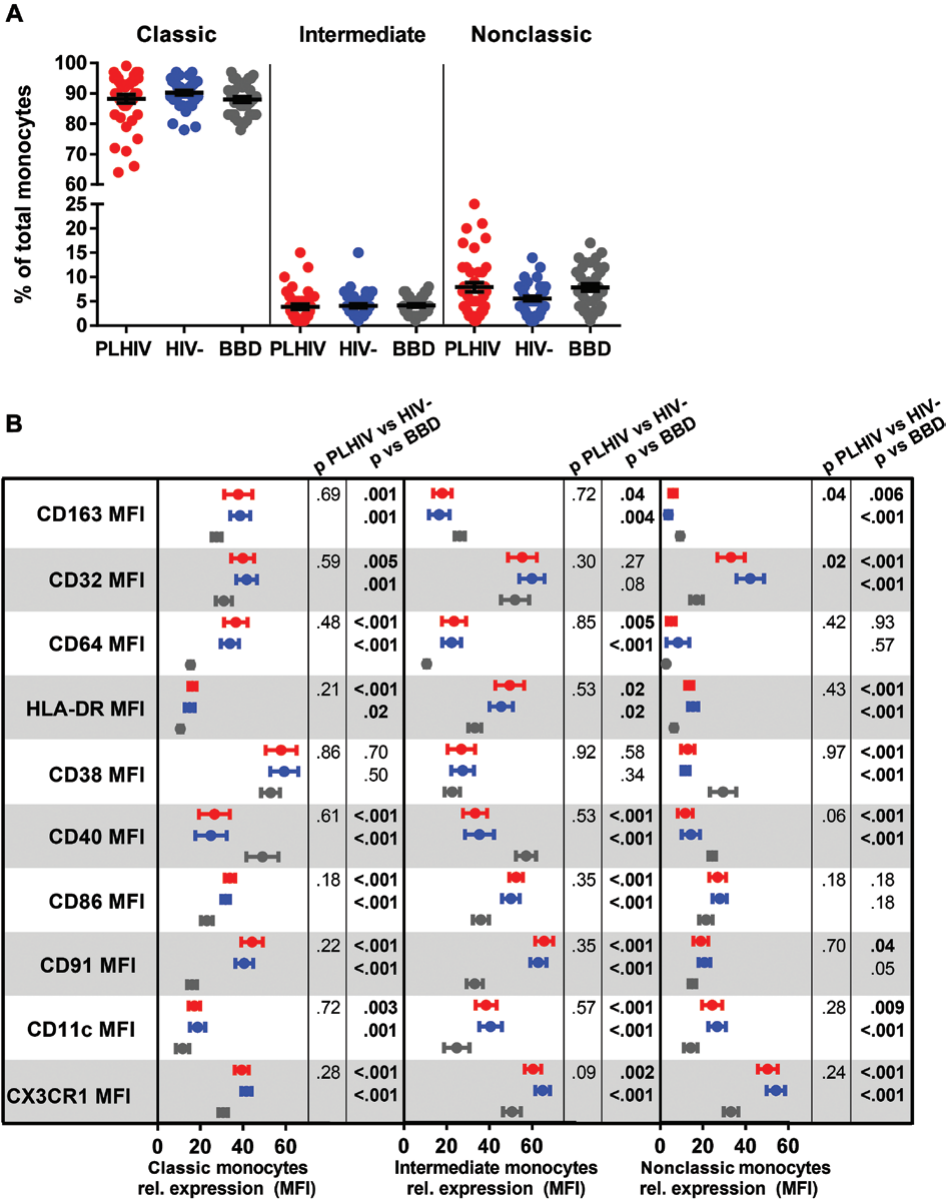
Phenotyping of monocytes from people living with human immunodeficiency virus (PLHIV), HIV-negative Comorbidity in Relation to AIDS (COBRA) participants and blood bank donors (BBD). (a) Percentages of classical (CD14^+^CD16^−^), intermediate (CD14^+^CD16^+^), and nonclassical (CD14^+^CD16^++^) monocytes of the total number of monocytes. (b) Relative expression of markers of activation (CD163, CD32, CD64, HLA-DR, and CD38), T-cell costimulation (CD40 and CD86), and adhesion (CD91, CD11c, and CX3CR1) molecules on classical, intermediate, and nonclassical monocytes. Mean fluorescence intensities (MFIs) were normalized to 100% over the classical, intermediate, and nonclassical monocyte subpopulations of PLHIV (red), HIV-negatives COBRA participants (blue), and BBD (gray) and represented as the mean and 95% confidence interval. Uncorrected *P* values assessed by multivariable linear regression corrected for age and gender are shown.

The percentages of classical (CD14^+^CD16^−^), intermediate (CD14^+^CD16^+^), and nonclassical (CD14^+^CD16^++^) monocytes did not differ significantly between PLHIV and HIV-negative COBRA participants. Furthermore, percentages of classical, intermediate, and nonclassical monocytes in BBD were compared with PLHIV and HIV-negative COBRA participants (Figure 1a). Monocyte subsets were analyzed for their expression of activation markers (CD163, CD32, CD64, HLA-DR, CD38), T-cell costimulatory molecules (CD40 and CD86), and adhesion molecules (CD91, CD11c, and CX3CR1). The expression of activation, costimulatory, and adhesion markers did not differ between PLHIV and HIV-negative COBRA participants, except for the expression of CD163 and CD32 (Figure 1b; Supplementary Table 1). CD163 expression was higher and CD32 expression was lower on nonclassical monocytes of PLHIV compared with HIV-negative COBRA participants (Figure 1b). Strikingly, both PLHIV and HIV-negative COBRA participants compared with BBD had distinct differences in the expression of monocyte activation and costimulatory and adhesion molecules. Compared with BBD, expression of CD163 was higher on classical monocytes and lower on intermediate and nonclassical monocytes of both groups of COBRA participants (Figure 1b). Expression of CD32 was higher on classical and nonclassical monocytes of COBRA participants, whereas expression of CD64 and CD86 was higher on both classical and intermediate monocytes of COBRA participants compared with BBD. Expression of HLA-DR, CD86, CD91, CD11c, and CX3CR1 was higher on all monocyte subpopulations of both PLHIV and HIV-negative COBRA participants compared with BBD (Figure 1b). Expression of CD38 was not different on classical and intermediate monocytes, but it was lower on nonclassical monocytes of PLHIV and HIV-negative COBRA participants compared with BBD. Expression of CD40 was lower on all monocyte subpopulations of PLHIV and HIV-negative COBRA participants compared with BBD (Figure 1b).

### Principal Components Analysis Identifies Distinct Monocyte Activation Patterns in People Living With Human Immunodeficiency Virus (HIV) and HIV-Negative Comorbidity in Relation to AIDS Participants

**Figure 2.**
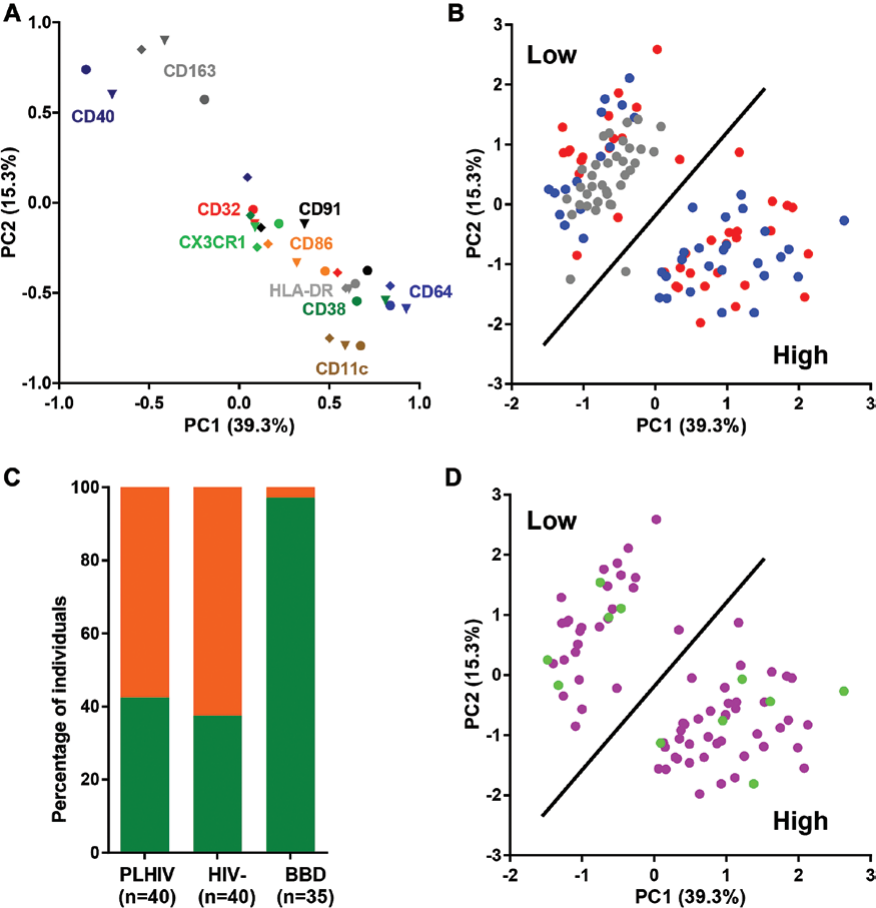
Principal component analysis of cellular markers of monocyte activation, costimulation, and adhesion data from people living with human immunodeficiency virus (PLHIV), HIV-negative Comorbidity in Relation to AIDS (COBRA) participants, and blood bank donors (BBD). (a) Loadings plot showing the relationship between the first 2 principal components (PC1, PC2) and the expression of CD163, CD32, CD64, HLA-DR, CD38, CD40, CD86, CD91, CD11c, and CX3CR1 on classical (●), intermediate (▼), and nonclassical (◆) monocytes. Each symbol shows the contribution of an individual marker to PC1 and PC2: CD163, dark gray; CD32, red; CD64, blue; HLA-DR, gray; CD38, dark green; CD40, dark blue; CD86, orange; CD91, black; CD11c, taupe; CX3CR1, green. (b) PLHIV (red), HIV-negative COBRA participants (blue), and BBD (gray) plotted based on the first 2 extracted principal components. (c) Frequency of individuals with low (green) or high (orange) cellular monocyte activation in PLHIV, HIV-negative COBRA participants, and BBD. (d) Cytomegalovirus (CMV)-positive (purple) and CMV-negative (green) COBRA participants plotted based on the first 2 extracted principal components.

The first principal component (PC1) explained 39.3% of the variation in expression of monocyte activation and costimulation and adhesion markers, whereas the second (PC2) explained 15.3% of this variation. The loadings plot shows that the markers CD32, CD64, HLA-DR, CD38, CD86, CD91, and CX3CR1 largely cluster together, whereas separate clusters of CD11c and CD163 together with CD40 were observed (Figure 2a). The PC1 is negatively correlated with CD163 and CD40 expression, whereas it is positively associated with CD64, HLA-DR, CD38, and CD11c expression. On the other hand, PC2 shows a relatively similar but opposite pattern of associations. When PLHIV, HIV-negative COBRA participants, and BBD are plotted based on PC1 and PC2, 2 very distinct clusters are observed (Figure 2b). Cluster 1 consists of 34 BBD, 17 PLHIV, and 15 HIV-negative COBRA participants with a low score for PC1 and a high score for PC2 indicating a low level of monocyte activation, whereas cluster 2 consists mainly of PLHIV (*n* = 23) and HIV-negative COBRA participants (*n* = 25) with a high score for PC1 and a low score for PC2 indicating a high monocyte activation (Figure 2b). Although the frequency of individuals with high or low monocyte activation did not differ significantly between PLHIV and HIV-negative COBRA participants, all but 1 BBD were classified as having low monocyte activation (Figure 2c).

When the PCA was performed using data from only the COBRA participants, PC1 and PC2 explained 11.1% and 4.1%, respectively, of the variation in expression of monocyte activation, costimulation, and adhesion markers within COBRA. In this analysis, a similar distribution between participants with high and low monocyte activation based mostly on PC1 was observed (Supplementary Figure 1). Monocyte activation status in COBRA participants was not associated with CMV status (Figure 2d) and CMV IgG antibody titers, HBV, and HCV serostatus (data not shown).

### Systemic Markers of Immune Activation, Inflammation, Intestinal Damage, and Coagulation in People Living With Human Immunodeficiency Virus (HIV) and HIV-Negative Comorbidity in Relation to AIDS Participants

**Figure 3.**
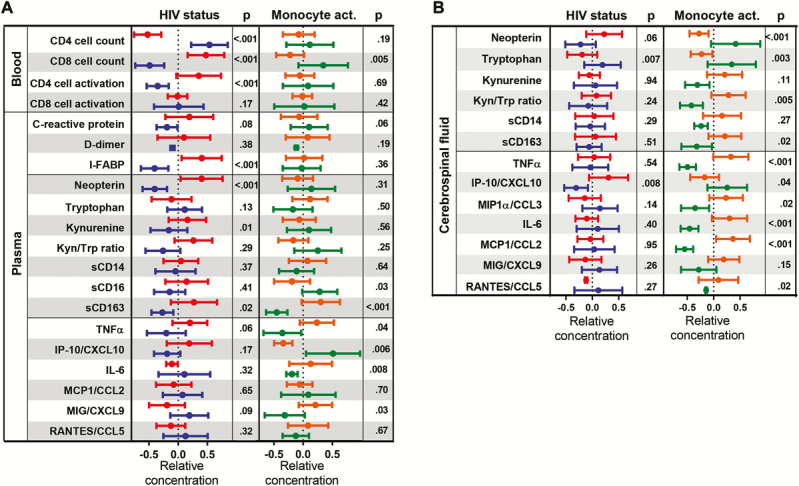
T-cell activation and soluble markers of coagulation, gut damage, monocyte activation, and inflammation in plasma and cerebrospinal fluid (CSF). (a) Systemic markers of monocyte activation (neopterin, tryptophan, kynurenine, soluble [s]CD14, sCD16, and sCD163), inflammation (C-reactive protein, TNFα, IP-10/CXCL10, IL-6, MCP1/CCL2, MIG/CXCL9, and RANTES/CCL5), intestinal damage (I-FABP), coagulation (D-dimer), and T-cell activation (%CD38^+^HLA-DR^+^ CD4 and CD8 T cells) in people living with human immunodeficiency virus (PLHIV) (red) and HIV-negative Comorbidity in Relation to AIDS (COBRA) participants (blue) and in all COBRA participants individuals with high (orange) or low (green) cellular monocyte activation. (b) Cerebrospinal fluid concentrations of monocyte activation (neopterin, tryptophan, kynurenine, sCD14, and sCD163) and inflammation (TNFα, IP-10/CXCL10, MIP1α/CCL3, IL-6, MCP1/CCL2, MIG/CXCL9, and RANTES/CCL5) markers in PLHIV (red) and HIV-negative COBRA participants (blue) and in all COBRA participants individuals with high (orange) or low (green) cellular monocyte activation. Data were normalized by Z-score normalization and represented as the mean and 95% confidence interval. Uncorrected *P* values assessed by multivariable linear regression corrected for age and gender are shown.

Concentrations of systemic markers of monocyte activation (neopterin, tryptophan, kynurenine, sCD14, sCD16, and sCD163), inflammation (C-reactive protein, TNFα, IP-10/CXCL10, IL-6, MCP1/CCL2, MIG/CXCL9, and RANTES/CCL5), intestinal damage (I-FABP), coagulation (D-dimer), as well as CD4 T-cell counts, CD8 T-cell counts, and proportions of activated T cells (%CD38^+^HLA-DR^+^ CD4 and CD8 T cells) were compared between PLHIV and HIV-negative COBRA participants and between individuals with high or low monocyte activation as determined by PCA. The PLHIV participants had lower CD4 T-cell counts, higher CD8 T-cell counts, higher concentrations of activated CD4^+^ T cells, higher plasma concentrations of the intestinal damage marker I-FABP, and higher monocyte activation markers neopterin, kynurenine, and sCD163 (Figure 3a; Supplementary Table 2). When the COBRA participants were classified based on their monocyte activation status, we observed that individuals with high monocyte activation had lower CD8 T-cell counts, higher systemic concentrations of sCD163, TNFα, IL-6, and MIG/CXCL9, but lower concentrations of sCD16 and IP-10/CXCL10 (Figure 3a; Supplementary Table 2). Monocyte activation status was not associated with lipid levels (total cholesterol, high-density lipoprotein cholesterol, low-density lipoprotein cholesterol, and triglycerides) in the COBRA participants (data not shown).

### Cerebrospinal Fluid Markers of Immune Activation and Inflammation in People Living With Human Immunodeficiency Virus (HIV) and HIV-Negative Comorbidity in Relation to AIDS Participants

In addition, we analyzed the concentrations of soluble monocyte activation (neopterin, tryptophan, kynurenine, sCD14 and sCD163) and inflammation (TNFα, IP-10/CXCL10, MIP1α/CCL3, IL-6, MCP1/CCL2, MIG/CXCL9, and RANTES/CCL5) markers in the CSF of COBRA participants. The PLHIV participants had lower tryptophan concentrations and higher IP-10/CXCL10 concentrations compared with HIV-negative participants (Figure 3b; Supplementary Table 3). Strikingly, a strong association was observed in COBRA participants (both PLHIV and HIV-negative) between peripheral cellular monocyte activation markers and soluble monocyte activation and inflammatory markers in the CSF (Figure 3b; Supplementary Table 3). Individuals with high cellular monocyte activation had lower concentrations of neopterin, tryptophan, and IP-10/CXCL10, a higher kynurenine/tryptophan ratio, and higher concentrations of sCD163, TNFα, MIP1α/CCL3, IL-6, MCP1/CCL2, and RANTES/CCL5 compared with individuals with low cellular monocyte activation.

## DISCUSSION

Systemic soluble markers of intestinal damage, innate immune activation, and inflammation such as I-FABP, sCD14, sCD163, indolamine 2,3-dioxygenase-1, IL-6, D-dimer, and CRP have been shown to be predictive for the increased morbidity and mortality observed in cART-treated PLHIV [18, 19]. In this study, cellular and soluble markers of monocyte activation, coagulation, intestinal damage, and immune activation were compared between PLHIV with suppressed plasma and CSF HIV-RNA on cART and 2 age-matched HIV-negative control groups: (1) selected HIV-negative individuals, recruited at sexual health clinics and who are comparable regarding most lifestyle and demographic factors; and (2) BBD, who were tested negative for HIV, HBV, HCV, syphilis, and HTLV infection and were at low risk for bloodborne infection. Confirming previous observations [9, 11], PLHIV participating in COBRA showed evidence of incomplete immune recovery, a damaged gut epithelial barrier, and high levels of monocyte activation, as reflected by lower CD4 counts, higher CD8 T-cell counts, a lower CD4/CD8 ratio, higher CD4^+^ T-cell activation, higher plasma concentrations of I-FABP, neopterin, and sCD163 compared with well matched HIV-negative COBRA participants. Furthermore, PLHIV had lower CSF concentrations of tryptophan, higher CSF concentrations of IP-10/CXCL10, and a trend towards higher CSF concentrations of neopterin, which is in line with previous studies [15, 16, 21]. Thus, our data indicate that despite virological suppression, PLHIV exhibit signs of ongoing immune activation in both plasma and CSF. However, we did not confirm higher plasma concentrations of sCD14, CRP, IL-6, MCP1/CCL2, MIG/CXCL9, and IP-10/CXCL10 [9, 12–14] or higher CSF concentrations of sCD14, sCD163, TNFα, IL-6, MCP1/CCL2, and MIP1α/CCL3 [17] in these virologically suppressed PLHIV. It should also be noted that in addition to a convenience sample of BBD, we also included a HIV-negative control group that was matched to the PLHIV for age and was comparable regarding most lifestyle and demographic factors; this may further explain why some previous observations were not confirmed. Furthermore, the PLHIV in our study are those who are considered to be successfully treated, as reflected by the relatively high CD4 counts, relatively high CD4/CD8 ratios, and apparent greater normalization of markers of chronic immune activation in their plasma and CSF, compared with other studies [9, 12–14, 17].

Comparison of the percentages of classical, intermediate, and nonclassical monocytes between PLHIV and both HIV-negative COBRA participants and BBD control groups revealed no differences, thereby confirming previous studies [22, 23]. Furthermore, no striking differences were observed in the expression of cellular monocyte markers between PLHIV and the selected HIV-negative COBRA participants. However, when either the PLHIV or HIV-negative COBRA participants were compared with the BBD, higher expression of markers of monocyte activation (CD163, CD32, CD64, and HLA-DR), costimulatory molecules (CD86), and adhesion molecules (CD91, CD11c, and CXCR1) were observed. This suggests that monocytes of both PLHIV and matched HIV-negative controls had a higher activation level compared with BBD. The lack of an appropriately matched HIV-negative control group in other studies may be one explanation for previously reported findings of an increased expression of activation markers on monocytes of virologically suppressed PLHIV compared with those in uninfected persons [9, 10]. Therefore, our findings reiterate the importance of including appropriate control groups when conducting immunological studies such as these.

In the Netherlands, BBD are tested negative for HBV, HCV, syphilis, and HTLV infection, and, in addition, are selected for their low risk of bloodborne infections based on a questionnaire regarding their general and sexual health, medication use, sexual risk behavior, and travel patterns. In contrast, the HIV-negative COBRA participants were recruited at sexual health clinics; due to lifestyle-related factors, these individuals are generally at higher risk for bloodborne infections than BBD. These differences in lifestyle-related factors may also increase their risk of exposure to (commensal) bacteria and viruses (eg, herpes viruses, papilloma viruses, polyomavirus) that trigger the innate immune system and thus may increase monocyte activation, without causing disease [24]. This is supported by the observation that CMV prevalence was higher in HIV-negative COBRA participants (77.5%) and lower in BBD (22.9%) than in the general Dutch population, 50% of whom are CMV positive at 50 years of age [25].

Principal components analysis of the cellular monocyte markers showed that PLHIV and HIV-negative COBRA participants could be separated further into 2 groups with either high or low monocyte activation [9, 26–28]. High expression of CD64, HLA-DR, CD38, and CD11c and low expression of CD163 and CD40 were associated with high cellular monocyte activation. The percentage of individuals with high monocyte activation did not differ between the PLHIV and HIV-negative COBRA participants; however, almost all BBD were classified as having low monocyte activation. Differential expression of the molecules assessed may well affect monocyte function and contribute to the immunological perturbations observed in these individuals [4]. High expression of Fcγ receptor 1 (CD64) may increase activation upon recognition of IgG and subsequent production of several proinflammatory cytokines such as TNF-α, IL-6, and granulocyte-macrophage colony-stimulating factor [27, 29]. High expression of CD11c, which is a receptor for complement factor iC3b, fibrinogen, intercellular adhesion molecule-1, and lipopolysaccharide and is involved in endothelial adhesion, may be reflective of an increased ability to migrate into tissues [30]. In addition, high monocyte activation was also associated with lower expression of CD163 and CD40. The hemoglobin scavenger receptor CD163 is exclusively expressed by monocytes and macrophages, and high expression is associated with the immunosuppressive M2c phenotype [31–34]. Extracellular Toll-like receptor activation of monocytes by bacteria or bacterial products results in shedding of CD163, which may be a mechanism to dampen acute and severe monocyte activation [35]. Furthermore, expression of CD40 on monocytes and dendritic cells is required to prime CD4^+^ T cells into a follicular homing phenotype via binding to CD40 ligand where they provide B cell help for effective antibody production [36, 37]. Low or absent CD40 expression may result in impaired antibody production and has been linked to immunodeficiencies characterized by defective Ig production [38].

High cellular monocyte activation in blood of PLHIV and HIV-negative COBRA participants was associated with high concentrations of inflammatory cytokines (TNFα, MIP1α/CCL3, IL-6, MCP1/CCL2, MIG/CXCL9, RANTES/CCL5) and monocyte activation markers (sCD163, tryptophan metabolism) in the CSF and, to a lesser extent, in plasma. We were surprised to find that high cellular monocyte activation was also associated with lower concentrations of sCD16 and IP-10/CXCL10 in plasma and lower concentrations of IP-10/CXCL10 and neopterin in CSF, because these are all considered monocyte activation markers [39–41]. Neopterin and IP-10/CXCL10 are both produced in response to interferon-γ [42, 43]. The lower concentrations of neopterin and IP-10/CXCL10 in plasma and CSF from individuals with high monocyte activation might be a reflection of diminished monocyte function in response to interferon-γ. Indeed, impaired monocyte function has been demonstrated previously [44, 45]. However, sCD16 and IP-10/CXCL10 can also be produced by other cell types (natural killer cells and neutrophils express CD16 and endothelial cells also produce IP-10/CXCL10) [42, 46], and therefore it cannot be excluded that sCD16 and IP/CXCL10 are reflective of other immunological processes, especially in individuals with low monocyte activation. This indicates that high cellular monocyte activation is predictive for a specific pattern of immune activation in the CSF, which is characterized by high concentrations of TNFα, MIP1α/CCL3, IL-6, MCP1/CCL2, MIG/CXCL9, RANTES/CCL5, sCD163, and tryptophan, but low concentrations of neopterin and IP-10/CXCL10.

Coinfection with viruses such as CMV, HBV, and HCV may contribute to the immune activation observed during treated HIV-infection [4, 47, 48]. We did not observe an association between HBV serostatus, HCV serostatus, CMV serostatus, or CMV IgG titers and the level of monocyte activation in COBRA participants. However, CMV infection was highly prevalent in PLHIV and HIV-negative COBRA participants, reflecting the high numbers of men who have sex with men in each group (for example, only 2 of the 40 PLHIV were CMV negative). In addition, the low prevalence of chronic HBV (*n* = 3) and HCV (*n* = 1) infection meant that we were unable to reliably assess the effects of coinfection with these viruses on monocyte activation.

High monocyte activation was not associated with high plasma concentrations of iFABP, a marker of intestinal damage, which suggests that microbial translocation is most likely not the main driver of the observed peripheral monocyte activation profile. However, we cannot exclude the possibility that peripheral monocyte activation is a reflection of lifestyle-related recent exposure to pathogens (bacterial or viral).

Although the cause of the peripheral monocyte activation profile remains unclear, the high expression of adhesion molecules suggest that these cells are more prone to leave the blood stream and move into tissues or cross the blood-brain barrier. Increased influx of peripheral monocytes into the CNS may account for the cytokine profile observed in the CSF and may subsequently also augment local immune activation. Moreover, cytokines such as TNFα and MCP1/CCL2 are known to reduce blood-brain barrier integrity, which could further enhance influx of peripheral monocytes into the CNS and thereby increase CNS immune activation [49].

A limitation of our study is its cross-sectional nature. Ongoing longitudinal follow-up will hopefully allow us to determine whether the increased monocyte activation is persistent and is related to ongoing (subclinical) or future pathologies. Furthermore, we cannot exclude the possibility that monocyte activation is a reflection of lifestyle-related exposure to other pathogens (unrelated to disease). Another limitation of our study is the small sample size, which restricts our ability to detect small differences between groups. The large number of statistical tests performed may also increase the type I error rate—we did not formally adjust *P* values to take account of multiple testing, but instead we used a PCA approach to identify a monocyte activation profile for each individual. This allowed us to reduce the dimension of our dataset, allowing us to focus primarily on differences in these profiles rather than on differences in expression of each of the markers of monocyte activation. Finally, our cohort mostly consists of white, northern European men—replication of our findings in other populations would be of value.

## CONCLUSIONS

In conclusion, markers of cellular monocyte activation did not differ between treated PLHIV and highly comparable, but HIV-negative individuals (COBRA) but were notably increased in both PLHIV and HIV-negative COBRA participants compared with BBD. More specifically, we were able to identify a subset of individuals within both the PLHIV and comparable HIV-negative COBRA participants with high levels of cellular monocyte activation, which was strongly associated with high proinflammatory cytokine production, and markers associated with an increased ability of monocytes to migrate into the tissues, and a decreased ability of monocytes to prime CD4^+^ T cells into a follicular homing phenotype. This shows that, in addition to HIV infection, there may be other (including lifestyle-related) factors, specific for individuals participating in the COBRA cohort, that drive cellular monocyte activation and inflammation and may thereby contribute to the comorbidities often seen in these individuals. Further studies are needed to determine whether high cellular monocyte activation and the subsequent high concentrations of proinflammatory cytokines relate to current or future neurocognitive or other comorbidity frequently observed in PLHIV [1–3]. Furthermore, we observed that HIV-negative COBRA participants, who were broadly comparable to PLHIV regarding most lifestyle and demographic factors, have greater immune activation compared with BBC. The potential clinical importance of this finding, particularly regarding future risk of complications associated with persistent immune activation, remains to be determined.

## Supplementary Data

Supplementary materials are available at *Open Forum Infectious Diseases* online. Consisting of data provided by the authors to benefit the reader, the posted materials are not copyedited and are the sole responsibility of the authors, so questions or comments should be addressed to the corresponding author.

## Supplementary Material

ofx108_suppl_Booiman_COBRA_Monocytes_Supplements_06052017
