# Supplementary material for: High Cellular Monocyte Activation in People Living With Human Immunodeficiency Virus on Combination Antiretroviral Therapy and Lifestyle-Matched Controls Is Associated With Greater Inflammation in Cerebrospinal Fluid
Source: Open Forum Infect Dis. 2017 May 25;4(3):ofx108. doi: 10.1093/ofid/ofx108 (PMC5494939; doi:10.1093/ofid/ofx108)
Supplement: ofx108_suppl_Booiman_COBRA_Monocytes_Supplements_06052017 [file ofx108_suppl_booiman_cobra_monocytes_supplements_06052017.doc]

**Supplementary Table 1.** Mean fluorescence intensity of activation, costimulation and adhesion markers on monocyte subsets.

|  |  | **PLHIV (n=40)** | **HIV-negative (n=40)** | **BBD (n=35)** |
| --- | --- | --- | --- | --- |
|  | Monocyte subset | Median (IQR) | Median (IQR) | Median (IQR) |
| **CD163 (MFI)** | classical | 2339 (1014-2867) | 2235 (1737-2786) | 1524 (1364-1822) |
|  | intermediate | 783 (403-1682) | 667 (223-1478) | 1346 (1222-1803) |
|  | non-classical | 196 (91-599) | 126 (63-218) | 518 (416-581) |
| **CD32 (MFI)** | classical | 5686 (3613-7883) | 5839 (4909-7945) | 4770 (3866-5465) |
|  | intermediate | 8210 (5865-10575) | 8938 (6951-10500) | 7568 (6243-9519) |
|  | non-classical | 4473 (2622-7424) | 6025 (3947-7566) | 2083 (1679-2821) |
| **CD64 (MFI)** | classical | 3759 (2126-4454) | 3446 (2013-4158) | 1519 (1268-1759) |
|  | intermediate | 1991 (872-2787) | 1911 (979-3069) | 1053 (830-1255) |
|  | non-classical | 286 (116-581) | 274 (134-784) | 257 (204-318) |
| **HLA-DR (MFI)** | classical | 14400 (12100-20050) | 13550 (9601-18800) | 10112 (8788-12290) |
|  | intermediate | 43350 (34450-64150) | 41900 (32800-53000) | 34693 (24659-40517) |
|  | non-classical | 13700 (9141-17500) | 14550 (9345-19450) | 6455 (4087-7803) |
| **CD38 (MFI)** | classical | 4083 (2595-5005) | 4324 (2882-4922) | 3789 (2847-4315) |
|  | intermediate | 1882 (486-2987) | 2048 (901-2800) | 1297 (1143-1897) |
|  | non-classical | 758 (472-1013) | 703 (569-1035) | 1575 (1140-2675) |
| **CD40 (MFI)** | classical | 205 (122-757) | 155 (94-681) | 631 (493-713) |
|  | intermediate | 427 (279-628) | 377 (302-788) | 797 (725-939) |
|  | non-classical | 117 (90-180) | 154 (125-220) | 352 (305-393) |
| **CD86 (MFI)** | classical | 1300 (1128-1611) | 1244 (1096-1436) | 989 (686-1153) |
|  | intermediate | 2001 (1773-2403) | 1939 (1741-2154) | 1438 (1187-1726) |
|  | non-classical | 1085 (727-1505) | 1111 (829-1449) | 816 (626-926) |
| **CD91 (MFI)** | classical | 5027 (3971-6239) | 4642 (3873-5802) | 1811 (1391-2478) |
|  | intermediate | 7352 (6439-8096) | 7167 (6021-7887) | 3693 (2656-4646) |
|  | non-classical | 2033 (1061-3287) | 2371 (1572-2904) | 1690 (1201-2122) |
| **CD11c (MFI)** | classical | 3080 (2238-4082) | 3328 (2153-4816) | 1954 (1561-2366) |
|  | intermediate | 7375 (5074-9448) | 7778 (5766-9787) | 4200 (3357-5280) |
|  | non-classical | 4410 (2324-7249) | 5630 (3094-6985) | 2435 (1949-3045) |
| **CX3CR1 (MFI)** | classical | 4790 (3903-5670) | 5063 (4658-5684) | 3685 (3311-4189) |
|  | intermediate | 7457 (6661-8313) | 7918 (7166-8755) | 6139 (5163-7117) |
|  | non-classical | 6320 (4907-7326) | 6734 (5568-8021) | 3676 (3076-4863) |

**Supplementary Table 2.** T cell counts and activation in blood, and soluble markers of coagulation, gut damage, monocyte activation and inflammation in plasma of COBRA participants.

|  | **HIV status** | |  | **Monocyte activation** | |
| --- | --- | --- | --- | --- | --- |
|  | **PLHIV (n=40)** | **HIV-negative (n=40)** |  | **High (n=48)** | **Low (n=32)** |
|  | Median (IQR) | Median (IQR) |  | Median (IQR) | Median (IQR) |
| **CD4 counts (cells/µl)** | 589 (470-800) | 961 (759-1233) |  | 755 (587-982) | 852 (576-1011) |
| **CD8 counts (cells/µl)** | 762 (636-1029) | 488 (364-621) |  | 557 (381-773) | 706 (412-845) |
| **Activated cells of CD4 (%)** | 2.58 (1.74-3.96) | 1.56 (1.03-2.57) |  | 1.94 (1.42-2.92) | 1.94 (0.95-3.92) |
| **Activated cells of CD8 (%)** | 7.46 (4.535-10.8) | 5.60 (3.29-9.65) |  | 7.15 (4.63-10.40) | 5.28 (3.33-10.14) |
| **C-reactive protein (mg/L)** | 1.75 (1.00-3.50) | 1.30 (0.50-2.70) |  | 1.30 (0.60-2.35) | 2.40 (0.95-4.35) |
| **D-dimer (μg/L)** | 277 (200-375) | 325 (225-433) |  | 295 (205-390) | 316 (204-427) |
| **I-FABP (ng/mL)** | 2.80 (2.10-3.75) | 1.51 (0.88-2.71) |  | 2.06 (1.30-3.34) | 2.35 (1.22-2.00) |
| **Neopterin (nmol/L)** | 8.82 (7.20-14.20) | 6.74 (5.64-7.50) |  | 7.38 (5.88-9.11) | 7.41 (6.02-11.82) |
| **Tryptophan (μmol/L)** | 65.97 (58.08-75.92) | 72.82 (62.29-81.93) |  | 71.55 (60.97-81.85) | 66.71 (58.07-77.04) |
| **Kynurenine (μmol/L** | 2.28 (1.98-2.57) | 2.00 (1.75-2.61) |  | 2.24 (1.75-2.56) | 2.05 (1.81-2.67) |
| **Kyn/Trp Ratio** | 33.29 (28.22-38.99) | 27.24 (23.40-33.50) |  | 30.46 (24.68-34.65) | 33.40 (15.91-39.26) |
| **sCD14 (ng/mL)** | 1151 (908-1441) | 1051 (928-1215) |  | 1099 (879-1340) | 1121 (947-1311) |
| **sCD163 (ng/mL)** | 1452 (1012-1948) | 1372 (859-1814) |  | 1116 (728-1534) | 1618 (1463-2060) |
| **TNFα (pg/mL)** | 269 (201-372) | 217 (165-287) |  | 291 (203-385) | 198 (149-248) |
| **IP-10/CXCL10 (pg/mL)** | 1.47 (0.76-1.81) | 0.90 (0.01-1.54) |  | 1.47 (0.79-1.85) | 0.76 (0.01-1.31) |
| **MIP1α/CCL3 (pg/mL)** | 93.02 (51.55-132.30) | 68.19 (46.45-102.63) |  | 66.78 (47.22-94.04) | 104.47 (63.40-178.69) |
| **IL-6 (pg/mL)** | 0.40 (0.01-1.48) | 0.71 (0.01-1.72) |  | 0.95 (0.25-1.85) | 0.01 (0.01-0.63) |
| **MCP1/CCL2 (pg/mL)** | 151 (120-189) | 163 (122-206) |  | 163 (131-187) | 155 (108-229) |
| **MIGCXCL9(pg/mL)** | 153 (131-235) | 225 (131-306) |  | 225 (153-252) | 131 (131-202) |
| **RANTES/CCL5 (pg/mL)** | 16829 (8498-46296) | 27788 (12431-49433) |  | 21090 (8498-52959) | 23157 (9720-45239) |

**Supplementary Table 3.** Soluble markers of monocyte activation and inflammation CSF of COBRA participants.

|  | **HIV status** | |  | **Monocyte activation** | |
| --- | --- | --- | --- | --- | --- |
|  | **PLHIV (n=40)** | **HIV-negative (n=40)** |  | **High (n=48)** | **Low (n=32)** |
|  | Median (IQR) | Median (IQR) |  | Median (IQR) | Median (IQR) |
| **Neopterin (nmol/L)** | 5.49 (4.65-8.71) | 4.91 (4.70-6.24) |  | 4.83 (4.58-5.49) | 6.97 (4.93-8.55) |
| **Tryptophan (μmol/L)** | 2.08 (1.77-2.44) | 2.37 (1.99-2.85) |  | 2.04 (1.80-2.42) | 2.46 (2.08-2.96) |
| **Kynurenine (μmol/L** | 0.135 (0.1-0.23) | 0.11 (0.1-0.2) |  | 0.16 (0.1-0.23) | 0.10 (0.10-0.17) |
| **Kyn/Trp Ratio** | 61.80 (27.78-111.20) | 38.15 (21.74-94.25) |  | 77.45 (30.33-110.30) | 28.59 (19.61-58.95) |
| **sCD14 (ng/mL)** | 93.16 (25.55-159.67) | 88.10 (52.89-120.84) |  | 88.23 (13.29-254.44) | 91.43 (66.45-119.31) |
| **sCD163 (ng/mL)** | 16.61 (8.30-27.48) | 14.62 (12.35-21.63) |  | 20.51 (13.90-26.23) | 11.87 (8.99-15.41) |
| **TNFα (pg/mL)** | 0.01 (0.01-0.65) | 0.01 (0.01-0.55) |  | 0.36 (0.01-0.79) | 0.01 (0.01-0.01) |
| **IP-10/CXCL10 (pg/mL)** | 228 (129-397) | 164 (108-226) |  | 158 (111-256) | 224 (163-329) |
| **MIP1α/CCL3 (pg/mL)** | 19.00 (19.00-72.04) | 51.45 (19.00-96.43) |  | 49.59 (19.00-96.43) | 19.00 (19.00-51.45) |
| **IL-6 (pg/mL)** | 1.81 (0.1-2.65) | 1.465 (0.825-2.865) |  | 2.10 (1.43-3.64) | 0.15 (0.10-1.81) |
| **MCP1/CCL2 (pg/mL)** | 484 (384-620) | 474 (407-557) |  | 530 (443-682) | 393 (338-476) |
| **MIGCXCL9(pg/mL)** | 3.09 (1.00-235) | 187.66 (1.00-270) |  | 153 (1.93-289) | 1.00 (1.00-225) |
| **RANTES/CCL5 (pg/mL)** | 0.01 (0.01-7.22) | 0.01 (0.01-13.25) |  | 0.17 (0.01-13.90) | 0.01 (0.01-0.30) |

Supplementary Figure 1:

**
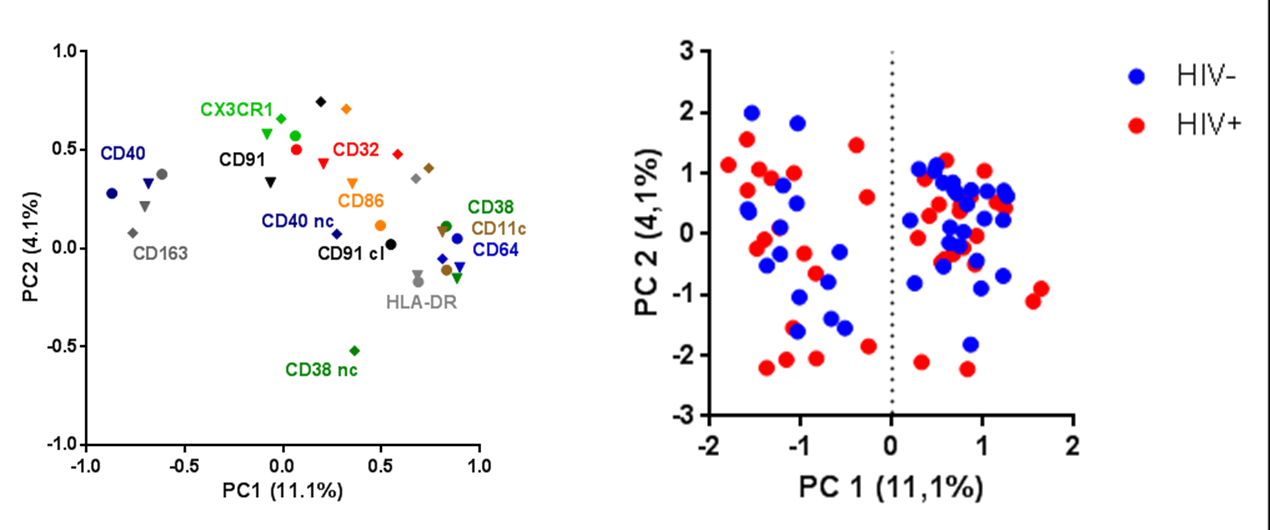
**

Supplementary figure 1: PCA analysis of HIV+ and HIV- participants of COBRA. Left: Loadings plot showing the relationship between the first two principal components and the expression of the different markers on classical (●), intermediate (⯆) and nonclassical (⬥) monocytes. Each symbol shows the contribution of an individual marker to PC1 and PC2: CD163, dark gray; CD32, red; CD64, blue; HLA-DR, gray; CD38, dark green; CD40, dark blue; CD86, orange; CD91, black; CD11c, taupe; CX3CR1, green. Right: HIV+ (red) and HIV- (blue) COBRA participants plotted based on the first two principal components.
